# Type 2 diabetes gene *TCF7L2 *polymorphism is not associated with fetal and postnatal growth in two birth cohort studies

**DOI:** 10.1186/1471-2350-10-67

**Published:** 2009-07-17

**Authors:** Dennis O Mook-Kanamori, Sandra WK de Kort, Cornelia M van Duijn, Andre G Uitterlinden, Albert Hofman, Henriëtte A Moll, Eric AP Steegers, Anita CS Hokken-Koelega, Vincent WV Jaddoe

**Affiliations:** 1 The Generation R Study Group, Erasmus Medical Center, Rotterdam, the Netherlands; 2 Department of Epidemiology, Erasmus Medical Center, Rotterdam, the Netherlands; 3 Department of Pediatrics, Erasmus Medical Center, Rotterdam, the Netherlands; 4 Department of Internal Medicine, Erasmus Medical Center, Rotterdam, the Netherlands; 5 Department of Obstetrics & Gynecology, Erasmus Medical Center, Rotterdam, the Netherlands

## Abstract

**Background:**

An inverse association between birth weight and the risk of developing type 2 diabetes (T2D) in adulthood has been reported. This association may be explained by common genetic variants related to insulin secretion and resistance, since insulin is the most important growth factor in fetal life. The objective of this study was to examine whether T2D gene polymorphism *TCF7L2 *rs7903146 is associated with growth patterns from fetal life until infancy.

**Methods:**

This study was performed in two independent birth cohort studies, one prospective population-based (Generation R), and one of subjects born small-for-gestational-age (SGA cohort). Fetal growth was assessed by ultrasounds in second and third trimesters of pregnancy in Generation R. Growth in infancy was assessed in both cohorts at birth and at 6, 12 and 24 months postnatally. *TCF7L2 *genotype was determined in 3,419 subjects in Generation R and in 566 subjects in the SGA cohort.

**Results:**

Minor allele frequency did not differ significantly (p = 0.47) between Generation R (T-allele: 28.7%) and the SGA cohort (T-allele: 29.8%). No differences at birth were found in gestational age or size (head circumference, length, weight) between the genotypes in either cohort. *TCF7L2 *genotype was also not associated with any pre- or postnatal growth characteristic in either Generation R or the SGA cohort.

**Conclusion:**

We found no evidence for an association between *TCF7L2 *genotype and fetal and early postnatal growth. Furthermore, this *TCF7L2 *polymorphism was not associated with an increased risk of SGA.

## Background

Several epidemiological studies have shown inverse associations between birth weight and metabolic diseases, including type 2 diabetes (T2D) in adulthood [1,2]. These associations may be influenced by common genetic variants [2]. Insulin is the most important fetal growth factor and insulin-mediated fetal growth might be affected by genetic polymorphisms that regulate fetal insulin secretion or insulin sensitivity [2]. Therefore, gene variants associated with T2D have been suggested as candidate genes for influencing early growth [2].

Genome-wide association (GWA) studies have consistently shown that the C>T substitution in *TCF7L2 *gene *(*rs7903146) increases the risk of T2D approximately 2-fold when two risk allele copies (TT) are present [3-5]. The T-allele of this *TCF7L2 *polymorphisms has been suggest to reduce proinsulin to insulin conversion [6], though the exact mechanism has not been elucidated yet. Other single nucleotide polymorphisms (SNPs) of the *TCF7L2 *gene have been shown to be associated with type 2 diabetes, although less strongly [7]. The T-allele of rs7903146, which according to HapMap has an allele frequency amongst Caucasians (CEU) of 28% [8], has been shown to be associated with reduced insulin response and secretion in both diabetic and non-diabetic individuals [9-11], though results in non-diabetics are not consistent [12]. This polymorphism may also lead to an increased risk of gestational diabetes [13]. Such findings make *TCF7L2 *one of the most important candidate genes for explaining the associations between low birth weight and T2D.

Freathy *et al*. were the first to investigate the association between *TCF7L2 *genotype and birth weight, and they found an association with maternal *TCF7L2 *genotype [14]. Each maternal copy of the risk allele was associated with a 30 grams increase in offspring birth weight, probably as a result of higher maternal glucose levels stimulating fetal insulin production [14]. After adjustment for maternal genotype, fetal *TCF7L2 *genotype did not influence fetal birth weight [14]. This finding was replicated in the Helsinki birth cohort [15]. In another study, no association was found between fetal *TCF7L2 *genotype and the risk of small size for gestational age [16]. Birth weight might be an inappropriate measure of the individual growth potential since different fetal growth rates may lead to the same birth weight [17]. Furthermore, rapid postnatal weight gain, especially in fat mass, has also been shown to be associated an increase risk of obesity and type 2 diabetes in later life, independent of birth weight [18,19].

Therefore we hypothesized that longitudinally measured fetal and postnatal growth are better parameters in the investigation of the possible effect of *TCF7L2 *on growth than specific growth characteristic such as birth weight. We first assessed the associations of *TCF7L2 *rs7903146 with fetal and postnatal growth characteristics in a population-based prospective cohort study among 3,419 subjects followed from early fetal life onwards. Second, we assessed associations of this genotype with birth weight and postnatal growth in 566 small-for-gestational-age (SGA) children participating in an independent cohort study.

## Methods

### Cohort Descriptions

#### The Generation R Study

The Generation R Study is a population based prospective cohort study from early fetal life onwards. The study is designed to identify early environmental and genetic determinants of growth, development and health from fetal life until young adulthood. It has been described previously in detail [20,21]. Fetal and postnatal growth and their main determinants were repeatedly measured by physical examinations, fetal ultrasounds, biological samples and questionnaires. We have previously shown that of all eligible children born in the study area 61% participated in the study [21]. The study has been approved by the Medical Ethics Committee of the Erasmus Medical Center, Rotterdam. Written informed consent was obtained from all participants or their parents.

#### Fetal growth and birth characteristics

Fetal ultrasound examinations were carried out during visits to one of the research centers. These fetal ultrasounds were used for establishing gestational age in the first trimester of pregnancy (conception to 12 weeks of gestational age), as well as for assessing fetal growth characteristics in second (17–25 weeks of gestational age) and third trimesters (> 25 weeks of gestational age) of pregnancy [22]. Fetal growth measurements used in the present study included head circumference (HC), abdominal circumference (AC) and femur length (FL) measured in second and third trimesters to the nearest mm using standardized ultrasound procedures [23]. Estimated fetal weight (EFW) was calculated by means of the formula from Hadlock using head circumference, abdominal circumference and femur length (log_10 _EFW = 1.5662 – 0.0108 (HC) + 0.0468 (AC) + 0.171 (FL) + 0.00034 (HC)^2 ^– 0.003685 (AC * FL)) [24]. First trimester ultrasound measures were not included for assessing growth characteristics because these ultrasound examinations were primarily performed to establish gestational age.

#### Birth and postnatal growth

Birth weight, date of birth and gender were obtained from community midwife and hospital registries. Information on head circumference or length at birth was not available, but many children were measured during the first two months of life. Well-trained staff in community health centers obtained postnatal growth characteristics using standardized procedures. Based on the routine health care program, the visits at which these growth characteristics were measured were grouped into three age periods: 6 months (range 5 to 8.99); 12 months (range 9 to 12.99); and 24 months (range 23 to 34.99 months). Postnatally, head circumference was not measured at the age of 24 months.

#### Population for analysis

**Figure 1:**
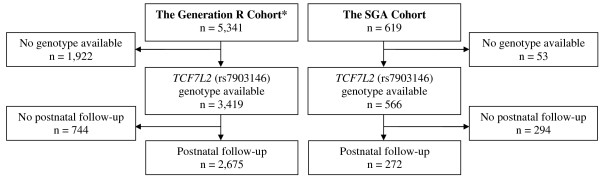
**Flow diagram indicating number of subjects in the two cohorts**. * All live-born, Caucasian, singleton subjects within Generation R.

Analyses were restricted to singletons from whom DNA was available for *TCF7L2 *genotyping and who also had Dutch or other Caucasian ethnicity as defined by having both parents born in the Netherlands or another European country (n = 3,419) (Figure 1). Fetal growth measurements were available for 3,320 and 3,384 children in second and third trimesters, respectively. Of these children, those living outside the study area postnatally (10%) were not followed up in infancy and a further 12% were lost during postnatal follow-up, leaving 2,675 subjects eligible for the postnatal analyses (Figure 1).

#### The SGA Cohort

The SGA cohort was designed for the purpose of assessing growth and development of subjects born SGA. Subjects were included at childhood age (n = 367) or at young adult age (n = 252). Children were included in the SGA cohort when they were SGA at birth, had short stature (height standard deviation score (SDS) for age and gender of below – 2 [25]), did not show catch-up growth in height, and had no growth failure caused by any other identified disorder. These inclusion criteria have previously been described [26]. Young adults included in the SGA cohort were randomly selected from hospitals in the Netherlands, where they had been registered because of being SGA. Only those young adults born at 36 weeks or more of gestation, being singleton and Caucasian and not suffering from conditions or receiving treatment known to interfere with growth, were invited to participate. SGA was defined as a birth length and/or birth weight SDS of below -2.0 for gestational age [27]. The Medical Ethics Committees of Erasmus Medical Center, Rotterdam, and of the participating centers approved all studies and written informed consent was obtained from all participants or their parents.

#### Birth and postnatal growth

Birth characteristics of the SGA cohort were collected from hospital registries. The gestational age of the subjects was determined by ultrasound in the first trimester, if available, and otherwise calculated from the date of the last menstruation. Growth data (head circumference, height and weight) measured during the first two years of life were collected from records of hospitals, community health services and general practitioners. Longitudinal growth data were available in 272 participants in the SGA cohort (Figure 1).

### Genotyping

DNA was collected from cord blood samples in the Generation R cohort and from peripheral venous blood samples in the SGA cohort. Cord blood for DNA isolation was available for 59% of all participating children of the Generation R cohort. When cord blood samples were missing, this result was mainly due to logistical constraints at the delivery. Venous blood samples were available in the complete SGA cohort. Genotyping of the C>T substitution in *TCF7L2 *(rs7903146) gene was performed using Taqman allelic discrimination assay (Applied Biosystems, Foster City, CA) and Abgene QPCR ROX mix (Abgene, Hamburg Germany). The genotyping reaction was amplified using the GeneAmp^® ^PCR system 9600 (95°C (15 minutes), with 40 cycles of 94°C (15 seconds) and 60°C (1 minute)). The fluorescence was detected on the 7900HT Fast Real-Time PCR System (Applied Biosystems) and individual genotypes were determined using SDS software (version 2.3, Applied Biosystems). Genotyping was successful in 98% and 91% of the samples in the Generation R and SGA cohort, respectively. To confirm the accuracy of the genotyping results, 276 randomly selected samples from the Generation R Study were genotyped for a second time with the same method. The error rate was less than 1%. The frequency distribution in Generation R did not deviate from the Hardy-Weinberg equilibrium in subjects with Dutch ethnicity nor did it deviate in the SGA cohort.

### Data analysis

With sample sizes in the Generation R Study of 3,419 and 2,675 subjects for fetal and postnatal analyses respectively, and assuming a statistical power level (1 – β) of 0.80, a level of significance (α) of 0.05 and a variance of 1.0, we were able to detect differences in growth characteristics of 0.048 SDS and 0.054 SDS respectively. First, differences in allele distribution between children born SGA (from the SGA cohort) and non-SGA subjects (from Generation R) were assessed. Differences were calculated using the Chi-square test. Second, we examined the differences in birth characteristics between genotype groups with linear regression analyses assuming an additive model. Weight, length and head circumference at birth and at different ages were analyzed using gender and age adjusted standard deviation scores (SDS) [27,28]. Standard deviation scores were obtained using Dutch reference growth curves (Growth Analyser 3.0, Dutch Growth Research Foundation). For Generation R, we used the first length SDS and head circumference SDS measured after birth and before the second month of life, since these measurements were not available at birth. Third, we compared fetal (only Generation R) and postnatal characteristics between the genotypes with linear regression analyses. Finally, to assess longitudinally measured weight and length patterns from fetal life to infancy, we performed repeated measures regression analysis in both cohorts with weight and length from birth to 24 months as outcome variables. This regression technique takes the correlation of multiple measurements within one subject into account, assesses both the time-independent and time-dependent effect of *TCF7L2 *genotype on growth, and allows for incomplete outcome data [29]. In these models, genotype was included as both intercept and interaction with age. To account for (gestational) age at each specific measurement, these analyses were conducted with age-adjusted standard deviation scores. The models can be written as:



In this model, the term including 'β_0_' reflects the intercept and the term including 'β_1_' reflects the slope of growth (weight or length) per week for the reference group (CC genotype). The terms including 'β_2_' and 'β_3_' reflect the age independent growth differences in weight (and length) between the different categories of the *TCF7L2 *genotype respectively [30]. All models were unadjusted (all growth characteristics are age and gender adjusted SD scores) since population genotype distribution is assumed to be unrelated to covariates and the effect estimates were not materially affected by adjusting for maternal age, pre-pregnancy body mass index or parity [31]. The occurrence of gestational diabetes in the entire cohort was 0.6% and did not affect the effect estimates. Therefore, occurrence of gestational diabetes was not included in the analyses.

All effect estimates are presented with their 95% confidence interval (95% CI). Statistical analyses were performed using the Statistical Analysis System version 9.1.3 (SAS, Stata corporation, College Station, TX, USA), including the PROC MIXED module for unbalanced repeated measurements as well as the Statistical Package of Social Sciences version 15.0 for Windows (SPSS Inc, Chicago, IL, USA).

## Results

Subject characteristics of Generation R and SGA cohort are presented in Table 1. The minor allele frequency distributions did not differ significantly (p = 0.47) between non-SGA subjects (from Generation R) (T-allele: 28.7%) and the SGA cohort (T-allele: 29.8%) (Table 2).

**Table 1:** Subject characteristics by cohort.

**Characteristics**	**Generation R**	**The SGA cohort**
Gender (% boys)	50.8%	47.2%
Gestational age (weeks)	40.1 (36.7 – 42.4)	38.0 (29.9 – 41.0)
Birth weight (grams)	3513 (511)	1819 (716)
Premature (gestational age < 37 weeks) (%)	2.9%	44.9%
Birth weight < 2500 grams (%)	2.5%	82.7%
Small for gestational age (weight <-2 SDS) (%)	0.9%	100%
Gestational diabetes (%)	0.6%	N/A

Values are means (SD), medians (95% range) or percentages. N/A = not available

**Table 2:** Distribution of TCF7L2 rs7903146 minor allele frequency according to cohort.

	**Allele frequency**		
	**C-Allele**	**T-Allele**	**p-value**

Non-SGA (Generation R) (%)	4828 (71.3)	1948 (28.7)	
SGA (SGA cohort) (%)	795 (70.2)	337 (29.8)	0.49

Non-SGA = All subjects from Generation R, excluding SGA subjects

SGA = birth weight SDS and/or birth length SDS <-2

p-value express differences in distribution between SGA and General population tested with Chi-square test.

No significant differences between genotype groups were observed in fetal growth characteristics in Generation R (Table 3). No differences in birth characteristics (head circumference, length and weight), between genotype groups were observed in either cohort (Table 4). Postnatal growth characteristics for both cohorts are shown in Table 5. No significant differences were found in either cohort for head circumference, weight or height at any age.

**Table 3:** Fetal characteristics according to fetal TCF7L2 rs7903146 genotype in the Generation R study.

	**CC** **(n = 1736)**	**CT** **(n = 1329)**	**TT** **(n = 301)**	**p-value^#^**
**Fetal characteristics second trimester**				
Head circumference (SDS)	0.04 (1.0)	0.02 (1.0)	0.05 (0.9)	0.88
Femur length (SDS)	-0.01 (1.0)	-0.01 (1.0)	0.05 (0.9)	0.64
Estimated fetal weight (SDS)	-0.06 (1.0)	-0.07 (1.0)	0.00 (1.0)	0.57
**Fetal characteristics third trimester**				
Head circumference (SDS)	0.11 (1.0)	0.13 (1.0)	0.15 (0.9)	0.45
Femur length (SDS)	0.01 (1.0)	-0.02 (1.0)	-0.04 (1.0)	0.34
Estimated fetal weight (SDS)	0.12 (1.0)	0.14 (1.0)	0.11 (0.9)	0.99

Values are means (SD). SDS = standard deviation score for age and gender.

^# ^p-values for additive models. Differences were tested using linear regression analyses.

**Table 4:** Birth characteristics in both cohorts according to *TCF7L2 *rs7903146 genotype of child.

**Generation R**		**CC** **(n = 1762)**	**CT** **(n = 1351)**	**TT** **(n = 306)**	**p-value^#^**
Gestational age (weeks)	n = 3419	40.3 (36.7 – 42.3)	40.3 (36.6 – 42.4)	40.1 (37.1 – 42.6)	0.83
Birth head circumference (SDS)*	n = 2314	0.22 (0.9)	0.24 (0.9)	0.26 (0.9)	0.55
Birth length (SDS)*	n = 1959	-0.07 (1.0)	-0.08 (1.0)	0.00 (1.1)	0.66
Birth weight (SDS)	n = 3419	0.21 (1.0)	0.22 (1.0)	0.20 (1.0)	0.97

**SGA Cohort**		**CC** **(n = 270)**	**CT** **(n = 255)**	**TT** **(n = 41)**	**p-value**^#^

Gestational age (weeks)	n = 566	38.0 (28.6 – 42.0)	38.0 (28.6 – 41.0)	38.0 (29.0 – 42.0)	0.57
Birth head circumference (SDS)	n = 203	-1.51 (1.4)	-1.20 (1.6)	-1.31 (1.4)	0.32
Birth length (SDS)	n = 491	-3.11 (1.4)	-3.27 (1.5)	-3.02 (1.5)	0.41
Birth weight (SDS)	n = 566	-2.40 (1.0)	-2.46 (0.9)	-2.32 (0.9)	0.58

* Length and head circumference were measured in the first two months of after birth.

Values are means (SD) or medians (95% range). SDS = standard deviation score for age and gender.

^# ^p-values for additive models. Differences were tested using linear regression analyses.

**Table 5:** Postnatal characteristics at 6, 12, and 24 months according to TCF7L2 rs7903146 genotype.

**Generation R**		**CC** **(n = 1375)**	**CT** **(n = 1063)**	**TT** **(n = 237)**	**p-value^#^**
**6 months**	Head circumference (SDS)	-0.02 (0.93)	-0.03 (0.89)	-0.06 (0.91)	0.83
n = 2675	Height (SDS)	0.03 (0.91)	0.03 (0.90)	0.07 (0.93)	0.81
	Weight (SDS)	0.41 (0.96)	0.44 (0.95)	0.54 (0.99)	0.14
**12 months**	Head circumference (SDS)	0.00 (0.89)	-0.04 (0.94)	-0.03 (1.12)	0.54
n = 2559	Height (SDS)	-0.01 (0.90)	-0.05 (0.90)	-0.01 (0.90)	0.70
	Weight (SDS)	0.18 (0.98)	0.18 (0.99)	0.24 (1.00)	0.63
**24 months**	Height (SDS)	-0.19 (0.93)	-0.21 (0.89)	-0.18 (0.87)	0.82
n = 2445	Weight (SDS)	-0.11 (0.99)	-0.13 (1.00)	-0.09 (0.96)	0.87

**SGA cohort**		**CC** **(n = 143)**	**CT** **(n = 107)**	**TT** **(n = 22)**	

**6 months**	Head circumference (SDS)	-1.38 (0.92)	-1.36 (0.90)	-1.74 (1.04)	0.41
n = 272	Height (SDS)	-2.39 (1.37)	-2.43 (1.26)	-2.51 (1.55)	0.93
	Weight (SDS)	-2.18 (1.40)	-2.22 (1.26)	-2.37 (2.05)	0.86
**12 months**	Head circumference (SDS)	-1.21 (0.83)	-1.24 (0.88)	-1.72 (1.06)	0.16
n = 268	Height (SDS)	-2.25 (1.25)	-2.30 (1.06)	-2.30 (1.47)	0.94
	Weight (SDS)	-2.14 (1.41)	-2.25 (1.15)	-2.17 (1.89)	0.82
**24 months**	Head circumference (SDS)	-1.10 (0.82)	-1.13 (0.87)	-1.60 (1.06)	0.20
n = 244	Height (SDS)	-2.39 (1.24)	-2.47 (1.05)	-2.94 (1.04)	0.20
	Weight (SDS)	-2.19 (1.33)	-2.31 (1.21)	-3.06 (1.65)	0.04

Values expressed as mean (SD). SDS = standard deviation score for age and gender.

^# ^p-values for additive models. Differences were tested using linear regression.

Finally, no differences were found in weight growth rate (SDS/year) from birth until the age of 2 years in either Generation R or the SGA cohort. Compared to the CC genotype, differences were -0.014 (95% confidence interval (CI): -0.064, 0.036) SDS/year and -0.028 (95% CI: -0.057, 0.002) SDS/year, for the CT and TT genotype, respectively, in Generation R. In the SGA cohort, differences were -0.134 (95% CI: -0.376, 0.108) SDS/year and 0.002 (95% CI: -0.125, 0.129) SDS/year, for the CT and TT genotype, respectively, using the CC genotype as a reference. Similarly, no differences were found in height growth rate from birth to 2 years in either cohort (data not shown).

## Discussion

In the current study, we found that T2D gene polymorphism TCF7L2 rs7903146 is not associated with growth in fetal life in the general population or with growth in early postnatal life in either the general population or in a cohort of subjects born SGA. We also confirmed previous suggestions that this variant of TCF7L2 is not associated with birth weight and, more importantly, demonstrated that it does not influence the fetal development using direct fetal measurements. Finally, we showed that this polymorphism does not appear to be associated with the risk of being born SGA.

To our knowledge, this study is the first to examine the association of *TCF7L2 *with longitudinally measured growth patterns in fetal and early postnatal life in two independent birth cohorts. In the Generation R Study, DNA for genotyping was available in 59% of all subjects and was isolated from cord-blood. Missing cord-blood was mainly caused by logistical restraints at delivery. Children who were not genotyped had a shorter gestational age (p < 0.001) and were lighter at birth (p < 0.001) than subjects who were genotyped. Of all genotyped eligible subjects at baseline, 22% did not participate in follow-up measurements. In the SGA cohort, genotyping was successful in 91% of the subjects and longitudinally growth data were available in 48% of the cohort. Our effect estimates could be biased if the associations between genotypes and growth characteristics differed between those with and without postnatal growth data available. In the Generation R cohort, no differences were observed between children with and without postnatal growth measurements. In the SGA cohort the T-allele was slightly more frequent in subjects with postnatal growth measurements than in subjects without these measurements (p < 0.05). Finally, it could be possible that there is differential effect of genotype on growth according to availability of follow-up data. This bias would affect our estimates, though such a bias seems unlikely.

Several studies have investigated the effect of common genetic variants related to insulin action and secretion on early growth [14,15,32,33]. Of the initially identified T2D gene polymorphisms identified by the GWA, fetal *CDKAL1 *(rs7754840) and *HHEX *(rs1111875) genotype, and maternal *TCF7L2 *(rs7903146) genotype have been shown to affect birth weight. Pulizzi *et al*. demonstrated in the Helsinki Birth Cohort that fetal *TCF7L2 *genotype did not interact with birth weight to increase the risk of T2D in adulthood [15].*TCF7L2 *rs7903146 has been shown to have the strongest genetic effect on T2D and this result has been replicated in several studies [3-5]. Therefore, *TCF7L2 *is a very important candidate gene for explaining the association between low birth weight and T2D risk. Our study is the first to investigate the effect of *TCF7L2 *rs7903146 on longitudinal growth in early life. Longitudinal assessment of growth provides more information than just measurements at birth as we have demonstrated earlier that different fetal growth patterns may result in a similar birth weight [17]. Furthermore, most SGA born children have catch-up growth during the first months of life but 15% remain small [34]. Thus, to investigate whether *TCF7L2 *rs7903146 influences fetal and postnatal growth, longitudinal growth data provide more information than birth weight alone.

Freathy *et al*. found an increase of birth weight for each fetal and maternal risk allele [14]. They concluded that the most likely mechanism for this association was that maternal genotype was associated with a reduction of maternal insulin secretion, leading to increased fetal glucose and insulin levels and subsequently increased birth weight, rather than a direct effect of the fetal genotype on birth weight. Pulizzi *et al*. found no effect of the fetal genotype of this polymorphism on birth weight. Since fetal and maternal genotypes are 50% correlated, it cannot be excluded that, when the risk allele is present in both mother and child, small effects of fetal genotype that reduce fetal growth could be masked by opposing effects of maternal genotype. Since maternal genotype was not available in our study, we were not able to test this hypothesis. However, we did not find any effect of fetal genotype on birth weight in the general population nor in a specific population of children with insufficient fetal growth resulting in small size for gestational age at birth. Our findings are therefore in line with the conclusions of these previous studies. Furthermore, we found no effect of fetal genotype on estimated fetal weight or weight during infancy, indicating that there is no evidence for any association between this fetal genotype and weight or change in weight during early life either. The effect of this polymorphism on the metabolic phenotype found in adults would therefore appear to develop after early childhood. Nonetheless, our results also could be explained by a lack of power and we cannot rule out that we were unable to detect smaller effects of this variant on early growth.

Regarding intra-uterine growth retardation, an earlier study examined the effect of *TCF7L2 *rs7903146 genotype on SGA. Cauchi *et al*. found no association between this genotype and SGA, using family-based association analyses in over 3,000 subjects of which 627 subjects were SGA [16]. In this analyses, the SGA group was slightly larger than in our current study and included parents, but postnatal growth data were not analyzed longitudinally. In our study, we did not find a difference in minor allele frequency between the general population (Generation R) and the SGA cohort. On the basis of two independent and negative studies, one may conclude that there is no association between this genetic polymorphism and risk of SGA.

## Conclusion

In summary, our results suggest that *TCF7L2 *rs7903146 does not influence growth from early fetal life to infancy. Furthermore, minor allele frequency was not different in SGA subjects than in non-SGA subjects, indicating that it is unlikely that this polymorphism is associated with the risk of being born SGA. Systematic searches for common genetic variants by means of genome-wide association studies will enable us to obtain a more complete understanding of which genes are involved in growth in fetal life and infancy.

## Competing interests

The authors declare that they have no competing interests.

## Authors' contributions

DOMK, SWKdK, ACSHK and VWVJ made substantial contributions to conception and design of the manuscript and the analysis and interpretation of data. CMvD and AGU were involved in the genetic analysis of the data. AH and HAM were involved in the design of the cohort. EAPS was responsible for the prenatal growth data collection. All authors were involved in drafting and revising the manuscript and have given final approval of the version to be published.

## Pre-publication history

The pre-publication history for this paper can be accessed here:


